# Beyond association: successes and challenges in linking non-coding genetic variation to functional consequences that modulate Alzheimer’s disease risk

**DOI:** 10.1186/s13024-021-00449-0

**Published:** 2021-04-21

**Authors:** Gloriia Novikova, Shea J. Andrews, Alan E. Renton, Edoardo Marcora

**Affiliations:** 1 Ronald M. Loeb Center for Alzheimer’s Disease, Department of Neuroscience, Icahn School of Medicine at Mount Sinai, New York, NY USA; 2 Department of Genetics and Genomic Sciences, Icahn School of Medicine at Mount Sinai, New York, NY USA

**Keywords:** Alzheimer’s disease, Myeloid cells, Functional genomics, Fine-mapping methods, Non-coding variants, Gene prioritization, Variant prioritization

## Abstract

Alzheimer’s disease (AD) is the most common type of dementia, affecting millions of people worldwide; however, no disease-modifying treatments are currently available. Genome-wide association studies (GWASs) have identified more than 40 loci associated with AD risk. However, most of the disease-associated variants reside in non-coding regions of the genome, making it difficult to elucidate how they affect disease susceptibility. Nonetheless, identification of the regulatory elements, genes, pathways and cell type/tissue(s) impacted by these variants to modulate AD risk is critical to our understanding of disease pathogenesis and ability to develop effective therapeutics. In this review, we provide an overview of the methods and approaches used in the field to identify the functional effects of AD risk variants in the causal path to disease risk modification as well as describe the most recent findings. We first discuss efforts in cell type/tissue prioritization followed by recent progress in candidate causal variant and gene nomination. We discuss statistical methods for fine-mapping as well as approaches that integrate multiple levels of evidence, such as epigenomic and transcriptomic data, to identify causal variants and risk mechanisms of AD-associated loci. Additionally, we discuss experimental approaches and data resources that will be needed to validate and further elucidate the effects of these variants and genes on biological pathways, cellular phenotypes and disease risk. Finally, we discuss future steps that need to be taken to ensure that AD GWAS functional mapping efforts lead to novel findings and bring us closer to finding effective treatments for this devastating disease.

## Background

### Introduction to the genetics of Alzheimer’s disease and genome-wide association studies

Alzheimer’s disease (AD) is the most common type of dementia among the elderly and, due to the aging global population, the number of people living with AD is expected to rise to 151 million worldwide by 2050 [1]. AD is characterized by progressive neurodegeneration that results in gradual cognitive and functional decline [2]. The neuropathological hallmarks of AD are the aggregation of amyloid-beta (Aβ) peptides into extracellular amyloid plaques and of hyperphosphorylated tau into intracellular neurofibrillary tangles [2]. In addition, reactive changes of astrocytes and microglia (gliosis) and accumulation of lipids (lipidosis) were also originally described by Alois Alzheimer and are often observed in disease [3].

The discovery of mutations in three genes (*APP*, *PSEN1* and *PSEN2*) that cause rare, early-onset and monogenic forms of AD led to the development of the amyloid cascade hypothesis, which postulates that abnormal aggregation and deposition of amyloid β peptide (Aβ is one of the products of APP cleavage by β- and ɣ-secretase, of which PSEN1/2 are two subunits) is neurotoxic and the primary cause of neurodegeneration [4]. Unfortunately, many clinical trials that relied on this hypothesis have failed to ameliorate AD, and no disease-modifying treatments are currently available [5], making it essential to generate novel therapeutic hypotheses for drug discovery that are based on the genetics of the common, late-onset forms of AD [6].

In contrast to monogenic forms of AD, over 95% of cases occur after the age of 65 and are generally considered to be oligogenic in nature [7]. Although more complex, this late-onset form of AD (LOAD) is also highly heritable [8]. Genome-wide association studies (GWASs) comparing clinically defined AD cases or AD-by-proxy cases (those with parental history of AD) with non-demented age-matched controls or AD-by-proxy controls, respectively, have been used to identify genetic variants associated with disease risk. A typical GWAS uses microarray genotyping to test the association of a single trait (e.g. disease diagnosis) or biometric phenotype (e.g., height, BMI, IQ) with a large number of common single nucleotide variants, also known as single nucleotide polymorphisms or SNPs, across the whole genome. These genotyped SNPs are selected because they each represent or ‘tag’ a group of genetic variants often inherited together as a haplotype block of high linkage disequilibrium (LD) spanning over a region of the genome. Thus, SNPs associated with traits by GWAS, rather than being interpreted as disease-causative variants in a given locus, should be thought of as identifying regions, or haplotypes, associated with disease. To date, AD GWASs have identified common genetic variants that modulate disease susceptibility in more than 40 genomic regions/loci [9]. However, these SNPs are mostly non-coding and the effect sizes of the identified associations are (with the exception of the *APOE* locus) very small. Together, this makes the identification of causal variants, genes, and molecular mechanisms underlying disease even more challenging.

In this review we will describe the recent advances and challenges in the identification of disease-relevant cell types(s) in which AD risk variants, genes and pathways likely operate. We then discuss variant and gene prioritization approaches that utilize statistical methods as well as integration of transcriptomic, epigenomic and chromatin interactions data. We will further describe experimental strategies to validate and further investigate downstream functional effects and risk mechanisms of candidate causal variants and genes. Finally, we will outline future directions in identifying AD risk variants and genes and investigating their downstream pathogenetic effects in cell and animal models.

In the next section we start by introducing the concept of functional mapping and why these efforts are critical for our understanding of AD pathogenesis. We will then discuss the concepts of molecular trait associations and their importance in deciphering the roles of non-coding disease risk variants.

### Introduction to functional mapping

Functional mapping is a set of approaches that aims to identify causal cell types, variants and genes in the region of association through various statistical methods and often integration of multiple sources of evidence, such as epigenomic, transcriptomic, and proteomic data. The overarching goal of fine-mapping is to quantify the strength of evidence that certain variants are not merely associated with the trait, but likely have functional impact in the relevant cell types and are responsible for modulating disease risk. Functional mapping is an essential component of post-GWAS analyses, since the etiology of the disease cannot be dissected without an understanding of the causal cell types, variants, genes, and their mechanisms of disease risk modification. However, there are many challenges associated with fine-mapping of GWAS loci.

First, most GWAS association signals contain a group of co-inherited variants that are in high LD with true causal variant(s). The LD patterns in a locus are affected by a multitude of factors, including recombination, natural selection and population bottlenecks, which can lead to complex haplotypes [10]. Hence, groups of variants with similar strength of disease associations as well as correlation with other haplotypes in the locus can make fine-mapping quite difficult. Second, some loci contain multiple independent signals associated with the phenotype, making it hard to identify candidate causal variant(s). While integration with molecular quantitative trait loci or molQTLs (associations between genetic variation and molecular traits, such as gene expression) can be used to prioritise causal variants, the abundance of such associations makes it difficult to assess the nature of the relationship between molecular phenotypes and disease associations [11]. Finally, non-coding variants identified through GWAS often exhibit their effects in a cell-type specific manner [12]. Hence, the success of fine-mapping strategies is dependent on the identification of the most relevant cell type and availability of relevant cell-type specific datasets.

Despite these challenges, fine-mapping efforts are critical in our understanding of genetic architecture and disease pathogenesis. The potential of GWAS to be translated into biological knowledge and clinical insight is directly dependent upon the effectiveness of fine-mapping efforts, which, when successful, identify candidate causal variants and their mechanisms of action and nominate candidate causal genes for further testing in human cells and mouse models, and as therapeutic targets for drug development and testing in clinical trials.

### Introduction to molecular trait and disease risk associations

When a haplotype is found to be associated with disease, the first critical step is to identify the functional variant(s), gene(s) and cell type(s) that mediate the statistical association of genetic variation at the locus with disease risk. As mentioned above, many disease-associated variants fall within non-coding regions of the genome. The function of these non-coding variants is often much less clear than those variants falling within gene coding regions, since there are a variety of ways by which non-coding variants can modulate disease susceptibility. These include effects on gene expression regulation, RNA splicing, and chromatin accessibility, among others, which in turn affect the biology of cells, tissues, organs, and systems in the causal path to disease [12].

In order to understand the functional consequences of genetic variation at disease-associated loci, genetic variation must first be linked to changes in molecular phenotypes. This is often done by treating given molecular phenotypes of interest, such as levels of gene expression (RNA or protein), RNA splicing, histone modifications that mark chromatin activity, or chromatin accessibility as quantitative traits in their own GWAS [13]. Genomic regions significantly associated with changes in the levels of these molecular phenotypes are then referred to as molecular quantitative trait loci (molQTLs). Gene expression associations (eQTLs) have been used to identify haplotypes that are associated with both disease risk and expression of certain genes, thus implicating them in disease pathogenesis [14]. Other molQTL associations can also be used to decipher the functional roles of non-coding variants, such as associations between genetic variation and splicing (sQTLs), histone marks (hQTLs), transcription factor binding (bQTLs), miRNA expression (miQTLs), DNA methylation (meQTLs), chromatin accessibility (caQTLs), and others [13]. Here, we will refer to these associations (i.e., associations between genetic variants and a trait) as GWAS associations.

**Fig. 1:**
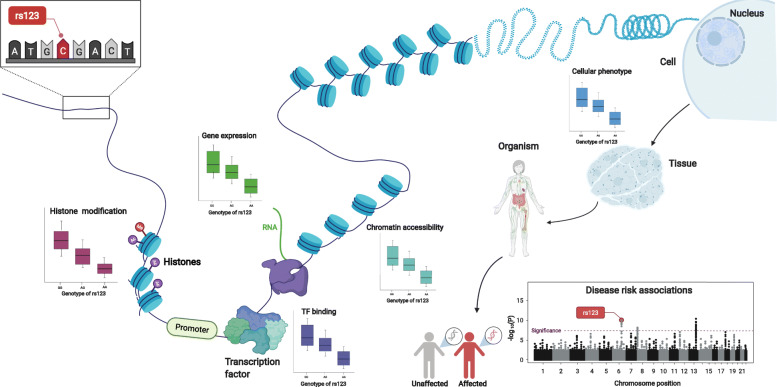
The figure depicts the effects non-coding disease associated variants can have on molecular and cellular phenotypes. These effects can be assessed through QTL analyses that identify significant associations between the dosage of the allele and various traits depicted, including histone modifications, transcription factor binding, chromatin accessibility and gene expression. These alterations finally lead to altered cellular phenotypes that subsequently translate to tissue level and organismal dysregulations and disease risk modification

Various statistical approaches have been developed to integrate multiple molecular associations with disease risk associations and thus gain insight into disease etiology. Integration of disease risk associations with other molecular trait associations, such as eQTLs, has been incredibly valuable in dissecting the likely mechanism of action of non-coding variants in disease risk loci. There are a multitude of methods that employ various statistical approaches to quantify the strength of evidence that disease risk is modulated by molecular phenotypes, such as chromatin activity and gene expression (Fig. 1). These methods generally help distinguish random colocalization of these signals from causality (e.g variant affects expression that in turn modulates disease risk) or pleiotropy (e.g a variant having distinct and parallel phenotypic effects on expression and disease risk). In this review, we will briefly discuss some of these methods before delving into their application to AD GWAS.

## Tissue and cell-type prioritization in AD

For many complex traits that are studied with GWAS, it is often unclear which cell types are causal for the development of the phenotype. Generally, it has been demonstrated that GWAS variants are enriched in DNase I hypersensitivity sites that mark open chromatin regions, suggesting these variants are likely regulatory [12].

However, regulatory regions are known to exhibit high cell-type specificity, making it critical to determine in which cell type(s) disease-associated variants are acting. This would facilitate prioritization of disease-relevant downstream target genes and selection of appropriate cell and animal models for functional analyses. Statistical methods that integrate GWAS variants with various tissue-specific and/or cell-type specific transcriptomic and epigenomic annotations have been developed to help prioritize the most relevant tissues and cell types.

One approach to assess the enrichment of a set of SNPs for regulatory activity in a given tissue- or cell-type is by testing if the proportion of disease risk variants residing in epigenomic annotations or being molQTLs in datasets from various tissues and cell-types is higher than the proportion of randomly selected SNPs which are also molQTLs or reside in active epigenomic annotations. This relatively simple approach was used to demonstrate enrichment of AD risk alleles in eQTLs of cells of the innate immune system (monocytes) as opposed to cells of the adaptive immune system (T lymphocytes) [15].

A different set of methods that relies on partitioning disease heritability (variation in phenotype that can be attributed to genetic variation in the population) across cell type-specific functional annotations have been recently developed and successfully applied to various complex traits [16, 17]. These methods attempt to quantify the proportion of disease heritability that can be attributed to various functional categories, such as cell-type specific epigenomic annotations [16]. Stratified LD Score Regression (S-LDSC) allows for the quantification of enrichment of functional annotations by estimating the proportion of genome-wide SNP heritability that is explained by the variants residing in those annotations [16], while accounting for LD and adjusting for annotation classes that are not specific to any cell type to ensure the specificity of the tissue or cell-type enrichment observed (e.g coding regions, UTR, histone marks and others )[16]. Using S-LDSC, our group has demonstrated the enrichment of AD risk alleles in myeloid-specific epigenomic annotations among all other available tissue and cell type-specific annotations, further implicating myeloid cell biology in the etiology of AD [18]. We and others have also recently reported that AD risk variants are enriched in active enhancer elements in myeloid cells (including human microglia) but not in other brain cell types [19], pointing to the activity of these regulatory elements as being likely affected by AD risk variation in a cell-type specific manner [18, 20].

When relevant functional annotations are not available, gene expression profiles can be leveraged using LDSC applied to genes specifically expressed in the tissue and/or cell type (LDSC-SEG), a method that was developed as an extension to LDSC [21]. This method tests if genome-wide SNP heritability is enriched in the regions surrounding the genes most specifically expressed in a tissue or cell type of interest [21]. LDSC-SEG has been recently used to demonstrate that AD heritability is also enriched in regions surrounding myeloid-specific genes [21]. RolyPoly is another method that can leverage not only bulk but also single-cell gene expression data to prioritize cell types relevant to the trait of interest through a polygenic modeling approach that allows for the possibility of multiple genes contributing to the trait [22]. It has been used to leverage brain single-cell expression dataset and identify AD relevant cell types, demonstrating a significant association between microglia and AD [22].

Rare-variant associations with AD have further reinforced the findings from common variant GWAS. AD genetic association studies interrogating whole genome, whole exome, and targeted datasets generated from familial and unrelated cohorts have identified several rare variants in protein-coding genes, such as *ABCA7, ABI3, PLCG2, SORL1* and *TREM2,* all of which are specifically or highly expressed in myeloid cells as compared to other brain cell types and play critical roles in the innate immune response, cholesterol metabolism, and endocytosis/phagocytosis [23–27]. Taken together, the findings from both common and rare variant GWAS point to the pivotal role of myeloid regulatory elements, genes and pathways in the etiology of AD.

## Gene prioritization in AD

### Colocalization of AD risk and molecular trait associations

The abundance of common variant associations with molecular traits, such as gene expression-related traits, increases the probability that colocalization with a disease risk association may occur at random. For example, about 18,262 protein-coding genes had at least 1 eQTL across all tissues tested in the GenotypeTissue Expression (GTEx) project, making it highly likely that a single-variant lookup might in reality be a false-positive [28]. Various methods have been adapted and developed to assess the likelihood that two GWAS associations are overlapping in a genomic region by chance.

Colocalization methods often integrate GWAS associations with eQTLs to assess the evidence that the same haplotypes underlie both disease risk and gene expression effects by utilizing various statistical tests instead of direct overlap or visual inspection of association signals. One of the most popular colocalization methods is *coloc*, a Bayesian method that tests the hypothesis that there is a single causal variant that underlies the association between two traits [29]. *Coloc* expresses the SNP causality in a region as a vector, where 1 denotes an associated variant and 0 denotes a variant that is not associated with at most one variant being considered associated with the trait at a time [29]. *Coloc* integrates over all possible configurations and computes probabilities of the data for each configuration [29]. These probabilities can then be summed and combined with the prior to access the support for each hypothesis tested [29]. To this end, coloc outputs five posterior probabilities where a large posterior probability for hypothesis three (PP3) indicating strong support for independent causal variants driving each trait, and a large posterior probability for hypothesis four (PP4) indicates strong support for a single variant driving both traits [29].

A recent example of application of coloc to a large GWAS of clinically diagnosed AD reported 31 prioritized genes across 13 loci in brain and blood/immune cell types [30]. The study nominated genes in previously reported loci such as *BIN1*, *SPI1*, *ABCA7, MS4A2* and *CD2AP* as well as genes in novel loci, including *KNOP1* in the *IQCK* locus [30]. A recent preprint reported the first eQTL map of human microglia [31]. Although the number of significant eQTLs was modest, coloc analysis identified multiple loci where an AD GWAS and microglial eQTL signals colocalized [32]. This approach nominated candidate genes whose expression in microglia is affected by AD risk alleles, including *BIN1* and *PICALM* [32]. Additionally, a recent study applied *coloc* to a large AD meta-analysis and found 391 colocalisations, nominating 80 candidate causal genes in 27 AD risk loci. This approach similarly identified previously nominated genes, such as *BIN1* and *PTK2B*, but also highlighted novel genes, including *TSPAN14* and *APH1 B *[33]**.** Finally, our recent work has utilized coloc to identify active chromatin regions whose hQTLs are colocalized with AD risk, hence identifying regulatory elements whose activity is modulated by AD risk variants. We identified fourteen active chromatin regions in human monocytes that are likely to be dysregulated by AD risk variants [20].

Although a powerful tool, coloc assumes that there is at most one association in each trait tested in the locus of interest; however, the tool can be used for loci with multiple independent associations [29]. This assumption can be tested using different methods that determine if there are multiple potentially independent statistical associations in the locus of interest. One such approach is performing conditional forward stepwise regression, which entails including the lead SNP as a covariate in a regression model and evaluating the presence of a residual signal. If significantly associated SNPs remain, some of them can be subsequently included as covariates until no residual signal is observed [10]. Since the phenomenon of multiple independent association signals is widespread, other frameworks, such as eCAVIAR, have been developed to account for the possibility of multiple causal variants at the locus [34].

In summary, colocalization analyses have been successfully utilized to identify non-random co-occurrence of GWAS associations, allowing for nomination of candidate regulatory elements and genes that might modulate disease risk.

### Exploring mediating effects of molecular phenotypes on AD risk

While coloc estimates the posterior probability of colocalization at one shared causal variant, another set of methods have been developed to more explicitly test the effects of gene expression on disease risk or any trait of interest [35]. Two widely used methods are transcriptome-wide association study (TWAS) and PrediXcan [35–39].

TWAS uses a panel of reference individuals to build models that can predict per-gene expression levels from genotypes at nearby variants, *cis*-SNPs, that reside within 1 MB of that gene [37]. These models are then used on a set of individuals with available genotype data to impute gene expression values [35, 37]. These values are then subsequently used to test for associations between gene expression and another phenotype measured in the same set of individuals (i.e., disease diagnosis )[35, 37]. This approach generates a set of significant gene expression-trait associations that can be interpreted as candidate causal genes in their respective loci [35, 37]).

TWAS has been successfully applied in the context of AD to identify associations between splicing QTLs and AD risk, thereby identifying likely causal genes in AD risk loci and proposing the mechanism of action of disease risk non-coding variants [40]. Specifically, Raj et al. conducted a TWAS study integrating intronic excision levels obtained from dorsolateral prefrontal cortex expression data with AD GWAS data and reported that alternative splicing is a likely mechanism of action in the *PICALM, CLU* and *PTK2B* loci [40].

Although TWAS offers a useful framework for nomination of candidate causal genes through expression-trait associations, there are several limitations that one needs to be aware of when examining loci that are significant in a TWAS study [36]. High inter-individual correlation in gene expression which can stem from co-regulation tends to lead to multiple significant hits in a locus. Additionally, since disease associated variants are often enriched in tissue-specific eQTLs, the use of disease-relevant tissue and/or cell type for TWAS is often recommended [14, 36]. It has previously been reported that the use of eQTL datasets from non disease-relevant tissues might lead to significant hits that exclude the causal gene(s) in the loci of interest ([14, 36]). Taken together, these findings suggest that selection of the tissue and/or cell type for TWAS analysis and interpretation of the results should be carefully done, taking into account the considerations mentioned above.

MetaXcan is a set of tools for integrating GWAS with other molecular GWAS associations to study the mediating effects of these phenotypes onto disease risk [38]. One of them, S-PrediXcan, allows for identification of associations between complex traits, such as gene expression and disease risk, using summary statistics. This is advantageous as it does not require individual level genotype data, which are often hard to obtain [38]. S-PrediXcan uses a similar gene expression imputation approach as TWAS, but implements a different statistical model [38]. TWAS uses Bayesian Sparse Linear Mixed Models (BSLMM) that include both sparse and polygenic components, allowing for a small set of predictor variants with large effect sizes as well as variants that contribute only a marginal effect to the overall prediction [38]. Given a largely sparse architecture of gene expression traits, PrediXcan avoids adding a polygenic component and only uses the sparse model [38, 41]**.** A recent study used an extension of PrediXcan, S-MultiXcan, that can improve the power to detect associations between complex traits by utilizing data from multiple tissues while accounting for cross-tissue correlation [42]. Using S-MultiXcan, Gerring et al. integrated eQTLs from GTEx and CommonMind Consortium (CMC) with AD GWAS data and identified 73 genes in GTEx and 12 genes in CMC whose expression was associated with AD [42].

Another method that allows us to study the causal or pleiotropic relationships between disease-based and molecular GWAS associations is Summary-data-based Mendelian Randomization (SMR), which has been successfully applied to AD GWAS. SMR is a form of instrumental variable analysis that exploits a natural experiment (i.e., genetic variants are assigned to individuals randomly at birth) to assess causal relationships between phenotypes [43]. This approach is akin to a randomized trial and utilizes variants that are correlated with an exposure (i.e., gene expression) to get insight into the relationship between that exposure and outcome (i.e., disease status )[43]. Specifically, SMR computes the effect size of a gene on a phenotype of interest by computing the ratio of the SNP effect sizes in GWAS and eQTL studies and further estimating its variance [44]. SMR also implements a method to distinguish between causality/pleiotropy and linkage through a heterogeneity in dependent instruments (HEIDI) test [44]. Unlike TWAS and S-PrediXcan, SMR does not use multiple SNPs to test for the association between a molecular trait and a disease phenotype, which can be considered as a disadvantage, but also provides an opportunity for selecting candidate causal SNPs by examining effect sizes of single SNP gene to trait associations [38, 45].

Marioni et al. have recently conducted a GWAS using self-reported parental history of AD (i.e., AD-by-proxy) and undertook SMR integrating with eQTL and meQTL data from dorsolateral prefrontal cortex, identifying highly significant associations with expression of *CR1*, *TOMM40* and *KAT8* amongst other genes [46]. Given the strong enrichment of AD risk variants in myeloid active enhancers, we took a myeloid-centric approach in using SMR to link identify putative causal associations between variant-harboring active chromatin regions and gene expression by integrating hQTLs with eQTLs [20]. This approach identified fourteen active chromatin regions likely to be dysregulated by AD risk variants and their putative target genes [20]. We then utilized SMR to subsequently identify significant gene expression and AD risk associations, mapping a path from myeloid enhancer activity to gene expression to disease risk modulation [20,47]. This approach allowed us to nominate candidate causal genes in twelve loci, including *BIN1*, *SPI1*, *ZYX*, *RABEP1* and *SPPL2 A* [20].

Taken together, the methods described above integrate multiple GWAS associations to begin deciphering the causal path between genetic variation, molecular phenotypes and disease risk. They have successfully been applied to AD, nominating candidate causal genes that likely modulate the risk for AD.

### Integration of epigenomic and chromatin interaction data for gene prioritization

Since common variants identified by GWAS rarely reside within coding sequences, epigenomic annotations of regulatory elements have become important for exploring the likely function of non-coding variants. Multiple large-scale consortia undertook an effort to generate epigenomic annotations, such as profiles of histone marks, chromatin accessibility, transcription factor binding sites and DNA methylation among others, in many cell types and tissues [48, 49]. These can be utilized not only for functional annotation of genetic variants, but also integrated with chromatin interaction assays. For example, HiChIP allows for identification of long-range interactions that are associated with the protein or histone mark of interest [50]. Another assay, promoter-capture Hi-C, uses biotinylated RNA oligomers that are complementary to all annotated promoters, thus revealing long-range promoter-interacting regions that can contain enhancers, silencers or other promoters [51]. These assays are extremely valuable in examining the likely roles of non-coding variants in mediating chromatin interactions and in identifying the genes that are likely regulated by the non-coding regulatory elements that harbor these variants and interact with target gene promoters. To this end, these data have been utilized in multiple functional mapping studies of AD.

We recently examined which genes are likely affected by myeloid active enhancers that harbor AD risk alleles by linking them to target genes through promoter-capture Hi-C and eQTL data from myeloid cells [20]. This approach identified candidate causal genes in 16 loci, including *AP4M1, BIN1*, *MS4A6A*, *PILRA*, *PTK2B* and *RABEP 1*[20]. Nott et al. took advantage of proximity ligation-assisted ChIP-seq (PLAC-seq) to generate chromatin interaction maps between active promoters (marked by H3K4me3) and distal regulatory elements in microglia [19]. By using ATAC-seq and PLAC-seq data in human microglia, regulatory elements containing AD risk variants were linked to their likely target genes, corroborating the evidence for many genes identified by our group [19]. Corces et al. generated chromatin interaction maps using HiChIP for H3K27ac, which marks both active enhancer and promoters, as well as single-cell chromatin accessibility maps in multiple brain regions to study the likely mechanisms of non-coding disease risk variants [52]. This identified multiple loci with microglia-specific open chromatin regions and nominated genes that are putatively affected by AD risk variants [52].

Combined these efforts demonstrate the utility of chromatin interaction data to provide insights into the chromatin architecture of AD risk loci and to nominate candidate causal genes in those loci.

## Variant prioritization in AD

### Bayesian fine-mapping for variant prioritization

One of the main goals of fine-mapping is to utilize the strength of the association of variants in the locus with the phenotype of interest, LD information and, molQTL and/or epigenomic data to pinpoint the most likely candidate causal variants. Various statistical methods for fine-mapping have been developed in recent years [10]. Heuristic approaches to fine-mapping usually involve considering SNPs with a certain level of correlation to the lead SNP, visually examining the LD in the locus and utilizing functional annotations to select strong candidate causal variants. This approach does not account for joint effects of variants on the phenotype of interest and lacks a systematic approach to evaluate the likelihood of the variant(s) to be causal. More robust approaches, such as Bayesian methods, utilize a statistical framework to estimate the posterior probability of each variant to be causal.

Bayesian fine-mapping tools, such as CAVIAR and PAINTOR, require GWAS summary statistics and an LD matrix ideally from the same individuals in which the GWAS was conducted [53–57]. If external LD reference panels are to be used to generate the LD matrix, the putative causal variants should be included in both summary statistics and an LD panel and the effect sizes of these variants should be the same in these populations [54]. Fine-mapping methods are sensitive to mismatches between GWAS data and LD panels, and can lead to inflated posterior probabilities if the LD panel is not derived from the same individuals or from a cohort with a very similar genetic composition [56]. Applying these methods results in posterior probabilities for each variant to be causal that can be used to prioritize variants for functional validation. Ranking posterior probabilities and setting a certain threshold ɑ for the sum of these posterior probabilities (e.g 95%) leads to the identification of credible sets, which are groups of variants that should contain causal variants(s) with the probability of ɑ [58].

CAVIAR was recently applied by Zhou et al. to fine-map the extended *APOE* locus and identify potential additional causal non-coding variants in the region that are *APOE*-ε4- independent [57,59]. Using this framework they identified nine non-coding variants in the *APOE* locus, which were associated with altered gene expression in *APOE* and other nearby genes in brain and blood [59].

PAINTOR is a Bayesian fine-mapping method that can incorporate functional annotations into the fine-mapping procedure [53]. In brief, PAINTOR gives higher weights to the variants that reside in certain functional annotations due to the fact that such localization makes it more likely to indeed be functional [55]. These weights are derived in an agnostic manner from the data itself with annotations that are highly enriched in GWAS loci of interest receiving higher weights [55]. Similar to other Bayesian fine-mapping tools, the output of PAINTOR is a posterior probability for each variant to be causal that can be further used in prioritization for follow-up functional studies [55].

We utilized PAINTOR in three loci (*BIN1, MS4A* and *ZYX*) using summary statistics and genotype data from the Alzheimer’s Disease Genetics Consortium (ADGC) case-control cohort as well as myeloid epigenomic annotations [20]. For the loci that were not significant in ADGC (but significant in larger AD GWAS) and were thus inappropriate for fine-mapping with PAINTOR, we developed an alternative strategy that prioritized disease-associated variants residing in enhancers in myeloid cells with strong evidence for potential disruption of binding motifs as well as an effect on gene expression [20]. Combined, these approaches allowed us to prioritize candidate causal variants and generate testable hypotheses about their likely mechanisms of action in seven AD risk loci [20]. Although larger fine-mapping studies of AD GWAS are needed, the availability of appropriate LD reference panels and genotype data from GWAS individuals as well as relevant epigenomic annotations and eQTL data from myeloid cells will be crucial for the success of these efforts.

### Variant annotation and functional impact prediction

Integration of epigenomic annotations to infer the likely functional impacts of non-coding variants has proven very fruitful in pinpointing candidate causal variants in disease-associated loci. For example, open chromatin regions, profiled with assays such as ATAC-seq, could point to variants that reside in nucleosome-depleted regions, are more likely to be regulatory and potentially disrupt binding of proteins in that region [60]. Profiling of histone modifications and transcription factor binding sites and integration of these data with the GWAS data could point to variants that reside within regulatory elements and give a hint about their function. For example, genomic regions enriched in the H3K4me3 mark usually point to gene promoters, H3K4me1 most often marks active and poised enhancers, while H3K27ac marks active enhancers and promoters [61]. Hence, localization of variants to these marks may suggest the type of regulatory element this variant is likely disrupting. Chromatin interaction assays can also be instrumental in identifying the targets (e.g., genes or other regulatory elements) of the regulatory elements in which disease-associated variants operate. These variant annotations can be used in simple heuristic fine-mapping approaches, where a group of variants are first selected based on their correlation with the lead SNP and further screened for their likely regulatory potential using available epigenomic and regulatory annotations. It should be noted that understanding the most likely causal cell type(s) and cell state(s) in the etiology of the disease of interest is critical to dissecting the regulatory potential of disease risk variants. In recent years databases, such as HaploReg and RegulomeDB, have integrated many omics datasets across a multitude of tissues and cell types to automate the variant annotation process, making it fairly straightforward to explore the group of variants for their likely regulatory potential [62 ,63].

Once variants are prioritized through these fine-mapping approaches, their likely mechanism of action needs to be identified. To this end, several approaches have been developed to predict the functional impact of a variant. One such approach is used by HaploReg, where variants are annotated for their effects on regulatory motifs through position weight matrices (PWMs )[64]. PWMs are then used to compute the change in log-odds score due to the variant that reflects the effect of this variant on the motif, i.e. disruption or creation of a binding motif [64]. Machine learning models have been developed to predict the impact of non-coding genetic variation on a range of epigenomic features [65, 66].

Taken together, the described approaches are important for understanding the likely regulatory elements (and their targets) that might be affected by the presence of disease-associated variants. These methods give insight into likely mechanisms underlying the statistical associations at disease risk loci and facilitate the generation of mechanistic hypotheses that can be further tested in functional experiments.

### Putting it all together: the successes and challenges of pinpointing candidate causal variant(s) in AD risk loci

The efforts described above nominated regulatory elements and AD candidate causal genes by studying the regulatory landscape of myeloid cells and integrating genetic, expression and chromatin data. Although these studies identify genes that can be subsequently studied through functional experiments and can give insight into common pathways affected by disease risk variants, they do not pinpoint candidate causal variants in disease risk loci. Non-Mendelian AD is a complex polygenic disease caused by dysregulation of entire biological networks due to risk variants with small to moderate effect sizes as opposed to single gene mutations with large effect sizes. Disruption of a transcription factor binding site by a non-coding disease risk variant can impact the activity of a regulatory element, leading to subsequent changes in chromatin conformation in the locus and altering interactions with other regulatory elements such as promoters, leading to changes in gene expression. Since regulatory elements like enhancers often interact with more than one promoter, genetic variation in these elements can lead to expression changes in multiple target genes (sometimes megabases away from the association signal) and other more complex downstream changes [67]. Thus, it is likely that disease-associated variants modulate disease risk by altering regulatory elements and in turn gene regulatory networks and gene expression programs in intricate ways, e.g., in specific cellular states or spatio-temporal patterns (e.g myeloid cells in the developing versus adult brain, or in the brain versus peripheral tissues like blood). Additionally, AD risk genes are likely to interact with each other and modulate disease risk by affecting biological pathways and cellular functions in complex ways. Hence, studying these non-coding variants in human cells and mouse models might provide greater insight into disease pathogenesis. To this end, understanding the functional impact of AD risk alleles in all disease-relevant contexts and at multiple scales (i.e., molecular and cellular) is critical to elucidate how they may impact disease susceptibility.

Previous studies have employed statistical fine-mapping approaches or integrated eQTL and epigenomic data to nominate candidate causal variants in AD risk loci. Recently Amlie-Wolf et al. have applied INFERNO, a method that integrates genomic annotations, transcription factor binding information and eQTLs from various tissues with GWAS summary statistics, to prioritize AD candidate functional variants [68]. Variants were prioritized by filtering for Approximate Bayes Factors (ABF) derived from Bayesian colocalization analyses with eQTLs, likely effects on transcription factor binding, enhancer overlaps and concordance between the tissue category of the colocalizing eQTL signal and the enhancer epigenomic annotation [68]. Collectively, the fine-mapping approaches in this study identified candidate causal variants in 10 regions with four genes (*EPHA1*, *CD33*, *BIN1* and *CD2AP*) being subsequently nominated for experimental validation [68].

In our recent work, we utilized a combination of heuristic and Bayesian fine-mapping approaches along with motif disruption predictions to prioritize candidate causal variants and generate hypotheses about their likely mechanisms of action in myeloid cells in seven AD risk loci [20]. For example, we identified a variant allele (rs636317-T) in the *MS4A* locus that likely disrupts an anchor CTCF binding site in the locus, likely leading to dysregulation in the local chromatin structure and increased expression of *MS4A4A* and *MS4A6 A *[20]. We also identified two alleles in the *BIN1* locus (rs6733839-T and rs13025717-T) that reside in PU.1 binding sites in a myeloid enhancer, disrupt motifs and likely binding of MEF2 and KLF transcription factors, leading to altered expression of *BIN 1 *[20].

Corces et al. developed a machine learning approach to score the effects of variants on chromatin accessibility, used additional complementary computational approaches and integrated these results with HiChIP data, colocalization analyses as well as information about transcription factor binding motifs [52]. This comprehensive approach resulted in the nomination of multiple AD risk variants, including the same variants in the *MS4A* and *BIN1* loci identified by our study described above [52].

Young et al. also nominated rs6733839-T in the *BIN1* locus as the likely AD risk-increasing allele using ATAC-seq data from primary human microglia and fine-mapping approaches [32]. The group reported a strong colocalization signal with microglial eQTL signal at the *BIN1* locus as well as significant allele-specific chromatin accessibility at rs673383 9[32].

## Functional validation of non-coding disease risk variants

Since non-coding variants in GWAS loci likely affect the activity of regulatory elements which in turn leads to dysregulation of gene expression programs, an important step in establishing the causal relationship between a non-coding variant and the associated trait is the subsequent experimental validation of bioinformatic predictions and further characterization of its functional impact on gene expression in disease-relevant cell types, states and contexts. Statistical methods described previously, including Bayesian fine-mapping and variant annotation, can provide robust hypotheses about the likely mechanism of the variant that can be evaluated in functional assays. If upstream fine-mapping analyses identify likely transcription factors bound at the variant site, allele-specific ChIP-quantitative PCR (qPCR) assays can be used to validate predicted preferential binding to a certain allele [69]. However in theory allele-specific qPCR tests the effect of the haplotype not a single variant.

Massively parallel reporter assays can identify enhancer activity- and expression-modulating non-coding variants, aiding in prioritization of likely functional candidate causal variants [70, 71]. Generally, these methods utilize a vector, containing a reporter gene (e.g., luciferase or GFP), a minimal promoter and a regulatory sequence containing the variant of interest inserted into the same plasmid [70]. After a plasmid library containing all variants to be tested is transfected into cells cultured in vitro, high throughput sequencing can be performed to determine the effects of the regulatory sequences on gene expression [70]. SNPs can be incorporated into these assays to study if there is a difference in gene expression between the alleles [70]. Although these assays do not reproduce in vivo cellular chromatin architecture, they can test a large number of variants before proceeding to more involved functional validation experiments.

Recent developments in genome editing tools, such as the CRISPR/Cas9 system, can be used to test the functional impact of altering a single variant. If upstream fine-mapping analyses offer a viable hypothesis of the variant’s mechanism of action, coupling single-nucleotide CRISPR-editing with functional readouts predicted to be altered, such as transcription factor binding, chromatin interactions, gene expression or other molecular and cellular phenotypes, can powerfully establish causality between the variant or a variant-harboring regulatory element and the trait of interest [45]. CRISPRa and CRISPRi systems have also been used to test the effect of regulatory elements, and a recently reported novel system, enCRISPRa and enCRISPRi, can alter enhancer-associated chromatin modifications, enabling investigation of relationships between regulatory elements and gene expression [72].

Taken together, the approaches described here, although non-exhaustive, can be very powerful in testing some of the regulatory elements and variants derived from fine-mapping procedures to validate and further characterize the functional impact of the disease-associated non-coding variants.

## Future directions

Although the studies that attempted fine-mapping of AD risk loci to date have converged on the same variants in multiple loci, including *BIN1* and *MS4A,* the majority of candidate functional variants in AD risk loci remain to be identified. There are many challenges that inhibit fine-mapping efforts of AD risk loci. Firstly, the largest GWAS studies in AD are large-scale meta-analyses, which makes gaining access to the genotype data from these individuals difficult. Since availability of appropriately large and representative LD panels for GWAS studies are critical for the success of statistical fine-mapping efforts, their lack can make fine-mapping efforts challenging [56]. Secondly, although previous studies have demonstrated a reproducible enrichment of AD risk alleles in myeloid epigenomic annotations and eQTLs, the specific myeloid cell type(s), cell state(s), cellular activity (ies) and spatio-temporal contexts in which they act to modify AD risk remain unclear. Recent findings indicate that the activation state of macrophages did not alter the enrichment of AD heritability in their epigenomic annotations, suggesting that AD risk alleles might be affecting core myeloid gene regulatory programs and cellular functions [73]. It has been reported that the loss of TREM2 affects the functions of macrophages in the central nervous system (CNS) and in macrophages in adipose and hepatic tissues during obesity, hinting that the AD-associated *TREM2* variants might act in multiple macrophage populations within and outside of the CNS, at baseline or in conditions of lipid overload and metabolic stress [74, 75].

There are, however, many exciting avenues that the fine-mapping effort of AD GWAS can undertake in the future. Firstly, as mentioned earlier, generation of more large-scale and fine-grained datasets with paired transcriptomic, epigenomic, chromatin interactions and genotype data in myeloid cells challenged with disease-relevant stimuli, such as lipid-rich cellular debris, will be essential to validate and expand current findings and nominate novel targets for experimental validation. Additionally, although AD risk alleles are reproducibly enriched in myeloid epigenomics annotations and regulatory effects, they are unlikely to explain the entirety of AD risk signals, highlighting the need for generating molQTL datasets from other brain and peripheral cell types, especially during aging and disease-relevant conditions. Secondly, GWAS data from non-European populations will undoubtedly be critical in the success of AD fine-mapping efforts [76]. Specifically, African populations are characterized by greater levels of genetic diversity, more extensive levels of population substructure and, most importantly, shorter LD blocks (partially due to more time allowed for recombination) than in non-African populations [77]. This creates an opportunity to leverage GWAS data from African and non-African populations to dissect disease-association signals with complex LD patterns and better fine-map these regions to pinpoint candidate causal variants. Hence, generation of additional molQTL and epigenomic datasets as well as expansion of AD case-control GWAS from individuals of African ancestry would be instrumental in deciphering the candidate causal genes and variants at AD risk loci. Thirdly, although in this review we focused on common non-coding variants, rare regulatory variation could also be explored in the context of AD and help explain some of the association signals. Finally, although human cells cultured in vitro represent valid and convenient systems for studying various molecular phenotypes, studying non-coding genetic variation in mouse models will allow for more in-depth evaluation of the role of these variants in myeloid and other cell types. A recent study by Mancuso et al. demonstrated that iPSC-derived microglia were transplanted into the mouse brain, successfully engrafting and transcriptionally resembling human ex-vivo microglia [78, 79]. This finding opens the door for implantation of engineered or patient-derived iPSC-derived microglia harboring causal variants or altered disease-relevant regulatory elements to examine their mechanism of action in vivo, in the aging and diseased brain. The hope is that the elucidation of genetic risk mechanisms at GWAS loci will aid the translation of genetic associations to novel drug targets and therapeutics as reviewed by Sierksma et al. [80].

## Conclusions

AD GWAS have identified more than 40 loci associated with the disease [9]. Functional mapping, e.g., identification of causal cell types, genes and variants, is an undertaking that is critical to our understanding of AD pathogenesis and ability to discover disease-modifying treatments. In this review, we discussed recent findings that pointed to the role of myeloid cells in the etiology of AD. Multiple studies highlighted the enrichment of AD risk alleles in myeloid epigenomic annotations (e.g., active enhancers), myeloid eQTLs as well as in genes specifically expressed in myeloid cells, including microglia [15, 18–21]. eQTL-based approaches, such as TWAS and SMR, demonstrated significant associations between AD risk and gene expression and nominated multiple candidate causal genes, many of which function within the endolysosomal compartment in myeloid cells [20]. These findings were also reinforced by integrative approaches that leveraged myeloid epigenomic annotations as well as chromatin interaction data to nominate candidate causal genes likely modulating AD risk. Finally, variant fine-mapping approaches resulted in identification of multiple candidate causal variants and dissection of their likely mechanisms of actions for further functional validation in cell and animal models.

Functional mapping has provided unprecedented insight into AD architecture by highlighting candidate causal variants, regulatory elements, genes, pathways and cell types that likely modulate disease risk. Performing larger GWAS in multiple ancestries coupled, the generation of more diverse, cell type- and cell state-specific datasets as well as ensuring that individual-level data or LD matrices are shared along with GWAS summary statistics in standard formats will propel these efforts further and help uncover disease risk mechanisms in more AD risk loci [81]. Follow-up functional studies to interrogate the roles of AD risk variants and genes in cells and animal models will be critical to shed light on the mechanisms of disease risk modification. Combined, these efforts will undoubtedly deepen our understanding of AD pathogenesis, bringing us closer to identification of disease-modifying treatments for this devastating disease.

## Data Availability

Not applicable.

## Abbreviations

AD: Alzheimer’s Disease
GWAS: Genome-wide Association Study
Aβ: Amyloid-beta
LOAD: Late-onset Alzheimer’s Disease
LD: Linkage Disequilibrium
OR: Odds Ratio
molQTL: Molecular Quantitative Trait Loci
eQTL: Expression Quantitative Trait Loci
sQTL: Splicing Quantitative Trait Loci.
hQTL: Histone Quantitative Trait Loci.
bQTL: Binding Quantitative Trait Loci.
miQTL: miRNA Expression Quantitative Trait Loci.
meQTL: DNA Methylation Quantitative Trait Loci.
caQTL: Chromatin Accessibility Quantitative Trait Loci.
S-LDSC: Stratified LD Score Regression.
LDSC-SEG: LD Score Regression Applied To Specifically Expressed Genes.
GTEx: Genotype-Tissue Expression Project.
PP3: Posterior Probability For Hypothesis 3.
PP4: Posterior Probability For Hypothesis 4.
TWAS: Transcriptome-wide Association Study.
CMC: CommonMind Consortium.
SMR: Summary-based Mendelian Randomization.
HEIDI: Heterogeneity In Dependent Instruments.
PLAC-seq: Proximity Ligation-assisted ChIP-seq.
PWM: Position Weight Matrix.
ABF: Approximate Bayes Factor.
qPCR: Quantitative Polymerase Chain Reaction.
CNS: Central Nervous System.
